# Primary surface rupture of the 1950 Tibet-Assam great earthquake along the eastern Himalayan front, India

**DOI:** 10.1038/s41598-017-05644-y

**Published:** 2017-07-14

**Authors:** Rao Singh Priyanka, R. Jayangondaperumal, Arjun Pandey, Rajeeb Lochan Mishra, Ishwar Singh, Ravi Bhushan, Pradeep Srivastava, S. Ramachandran, Chinmay Shah, Sumita Kedia, Arun Kumar Sharma, Gulam Rasool Bhat

**Affiliations:** 1 Wadia Institute of Himalayan Geology, Dehradun, India; 2 Geological Survey of India, SU: WB & AN, ER, Kolkata, India; 3 Physical Research Laboratory, Ahmedabad, India; 4 Centre for Development of Advanced Computing, Pune, India; 5 Department of Geology, Kumaun University, Nainital, India; 6 Department of Disaster Management, Pondicherry University, Andaman Campus, Port Blair, India

## Abstract

The pattern of strain accumulation and its release during earthquakes along the eastern Himalayan syntaxis is unclear due to its structural complexity and lack of primary surface signatures associated with large-to-great earthquakes. This led to a consensus that these earthquakes occurred on blind faults. Toward understanding this issue, palaeoseismic trenching was conducted across a ~3.1 m high fault scarp preserved along the mountain front at Pasighat (95.33°E, 28.07°N). Multi-proxy radiometric dating employed to the stratigraphic units and detrital charcoals obtained from the trench exposures provide chronological constraint on the discovered palaeoearthquake surface rupture clearly suggesting that the 15^th^ August, 1950 Tibet-Assam earthquake (*Mw* ~ 8.6) did break the eastern Himalayan front producing a co-seismic slip of 5.5 ± 0.7 meters. This study corroborates the first instance in using post-bomb radiogenic isotopes to help identify an earthquake rupture.

## Introduction

**Figure 1:**
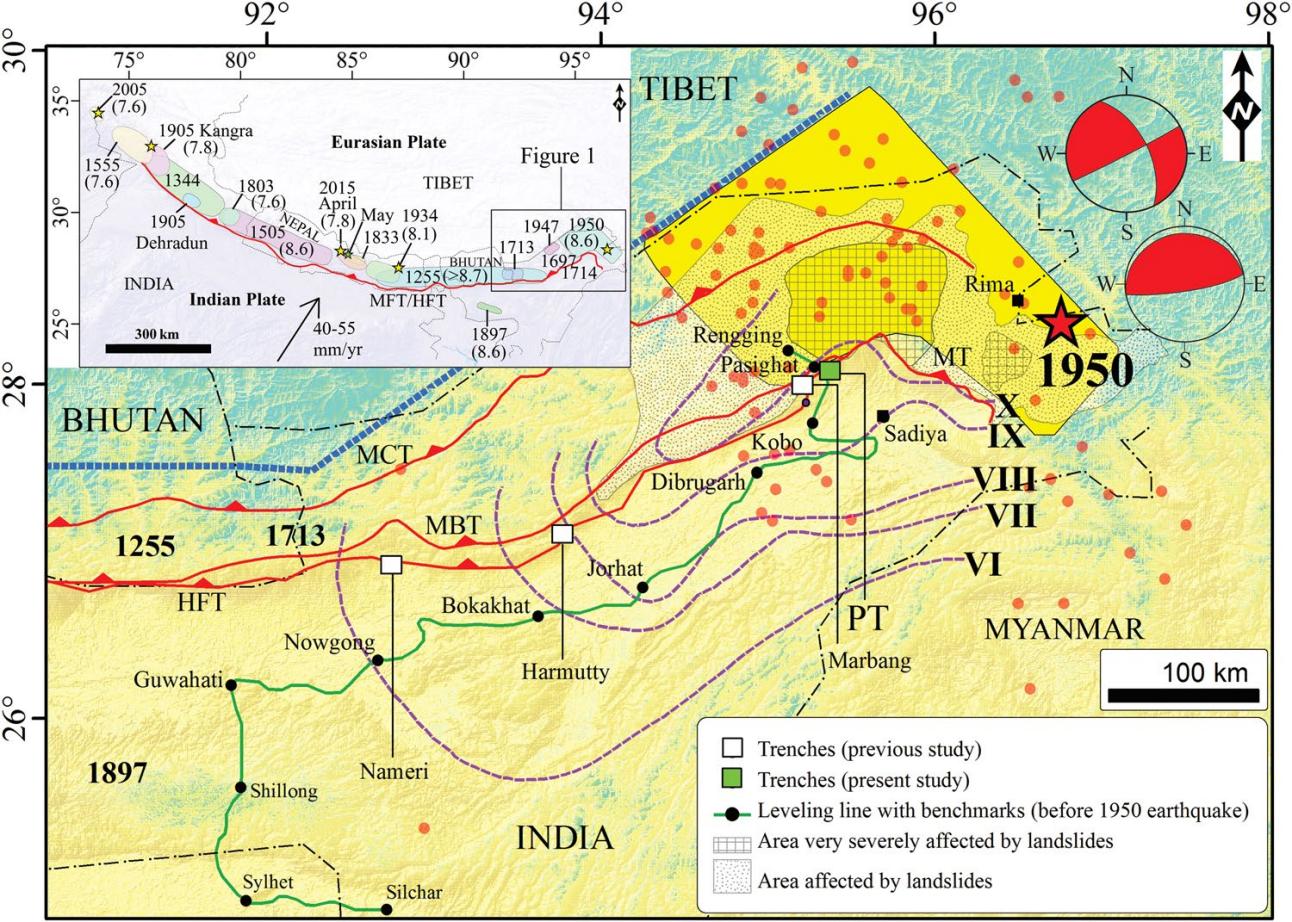
Map showing the historical earthquakes in the eastern Himalaya (1255, 1713, 1897, 1950-yellow polygon). 1950 intensity isoseismals (violet dotted lines denoted by Roman numerals) from previous study^35^. Previous trench locations in the eastern Himalaya (solid white squares)^4, 9^ and present study at Pasighat (solid green square); 1950 epicenter (red star) from previous study^15^. 1950 earthquake aftershocks (red dots), locking line at ~3.5 km elevation contour^17^ (blue dashed line), co-seismic landslides associated with 1950 earthquake^62^; leveling line^30^; international border (thin black dashed-dotted line). (**Inset**) Map showing the rupture zones of large-to-great earthquakes in the Himalaya adopted from previous studies^7, 14, 42^ and the convergence rate between India and Eurasia. Maps were prepared in Arc GIS v9.3 using SRTM GTOPO 30 m imagery available at http://glcfapp.glcf.umd.edu/data/srtm/description.shtml.

Continued convergence between India and Eurasia has produced large-to-great earthquakes along the ~2500 km long Himalayan front (Fig. 1 inset). As a clear elucidation of their rupture segments has remained enigmatic, recent palaeoseismic investigations have provided insight into the rupture dynamics and recurrence interval of these seismic events and the associated hazards^1–14^. Some dissonance between historical and palaeoseismic data exists due to lack of precise chronological constraints, and this lacunae makes it difficult to correlate surface rupture events through time. Occurrence of great devastating earthquakes like the A.D. 1934 and 1950 events were ascribed to blind faults that did not reach the surface. Contrastingly, a recent study did locate the 1934 earthquake surface rupture^11^. With this in view, it was quite unlikely that the 1950 earthquake (with seismic moment *M*_*o*_ = 4.0 × 10^28^ Dyne-cm and moment magnitude *M*_*w*_ = 8.6) being the largest known, did not produce any surface rupture^15^.

Palaeosesimic investigations close to the shaking zone of the 1950 event did not reveal any surface rupture that could be attributed to this earthquake^4, 9^. Aftershock pattern, fault plane solutions and co-seismic slip proposed in previous studies have been contradictory, variously suggesting a low-angle thrust faulting with oblique component^15^ and strike-slip faulting^16^ with two nodal planes NNW-SSE and ENE-WSW^16^. The former^15^ ascribed the thrust faulting parallel to the strike of the Himalayan Frontal Thrust (HFT), but the latter^16^ related it to the NS-NW striking Mishmi Thrust (MT). Furthermore, in eastern Himalaya the pattern of strain accumulation and its release is not simple due to greater locking width^17^ and structural complexity.

Moreover, the frontal thrust is linked with the Sumatra subduction zone^18–20^. Based on the fault plane solutions for shallow and intermediate earthquakes and Landsat imagery interpretation from Burma and the surrounding regions, it was suggested that the Indian Plate rotated several degrees clockwise to penetrate into Eurasia past Burma^18^. The Himalayan arc meets the Indo-Burmese ranges at the eastern syntaxis, which is in turn attached to the Bengal Basin. The Bay of Bengal basin subducts beneath the Sumatra Subduction Zone^20^. A general issue which remains enigmatic is whether great earthquakes like the 1934 (*M*_*w*_ ~ 8.4) and 1950 (*M*_*w*_ ~ 8.6) release all the hitherto stored energy (generated by Indo-Tibet convergence), or only a portion of the elastic strain accumulated during the interseismic period. Due to this problem, the recurrence time of such events remains undecipherable. Unavailability of surface rupture enhances the threat of a potential earthquake and its seismic hazard. However, a surface rupture as well as blind fault related earthquake mitigates the potential hazard associated with a future earthquake by releasing the stored strain energy either partly or fully. Therefore, the style and rate of seismic strain accumulation and release through the 1950 earthquake has remained unclear^21^ along this knee-bend plate boundary system which forms a complex triple junction joining the Indian and Eurasian plates with the northern end of the Burma platelet^22, 23^. Such intricacies led us to further explore for evidence of surface rupture within the region of strong shaking of the 1950 earthquake (Fig. 1) and help understand the deformation and assessment of the seismic hazard associated with the easternmost segment of the Himalaya with substantive human and infrastructural dimensions.

## Geomorphic Uniqueness of the Study Area

**Figure 2:**
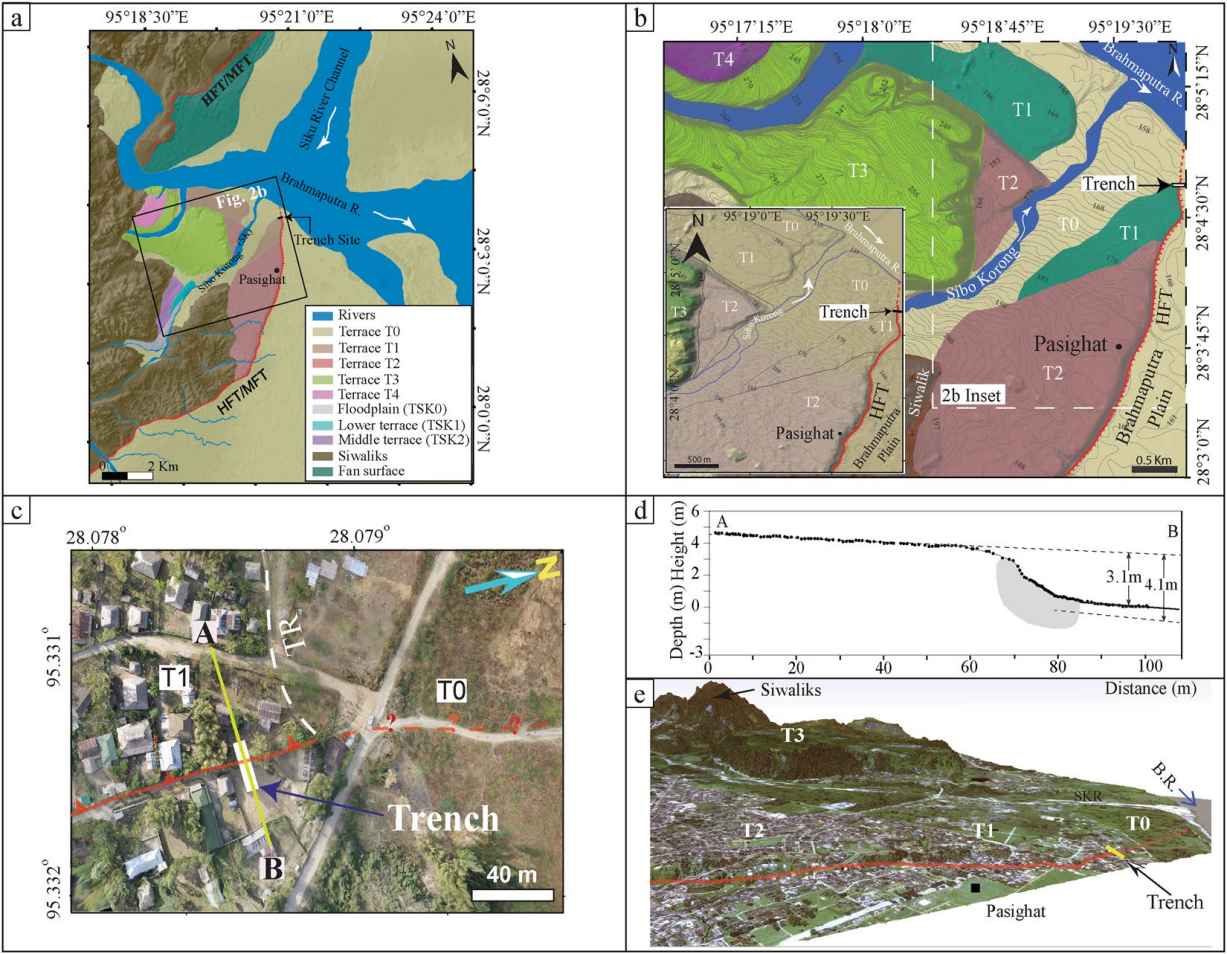
(**a**) Regional tectono-geomorphic map of the trench site at Pasighat prepared from Cartosat-1 satellite imagery. (**b**) Geomorphic map of Pasighat area showing different features mapped in the field aided with Pleiades satellite imagery. Inset map showing the enlarged view of the trench site. Maps were prepared using Pleiades and Cartosat-1 imageries purchased from http://www.nrsc.gov.in (Source: NRSC, ISRO/DOS) in SOCET GXP version 4.1.0 software and artwork in Adobe Illustrator CS5 software. (**c**) Figure showing the aerial photo-mosaic of specified view of the Pasighat trench site obtained using Ricoh GR 8.3 megapixel digital camera mounted at the bottom of UAV (DJI Phantom 1 multi-rotor cod copter); for field photograph of the trench site refer to Fig. S1a; mosaic was made using AgiSoft Photo Scan Pro software purchased from http://www.agisoft.com (**d**) RTK-GPS profile of the Pasighat scarp showing the excavated trench (grey polygon) prepared in Leica Geo Office v7.0 programme and artwork in Adobe Illustrator CS5 software; Scarp height is 3.1 m (when measured as the vertical difference between the sloping profile across the scarp) and 4.1 m (when measured as the vertical separation between the sloping profile of the hangingwall and the event horizon) in the footwall. Location of profile A-B is shown in Fig. 2c. Microtopographic map of the trench site is provided in Supplementary Figure S1d. (**e**) 3D perspective view of the Pasighat trench site generated using Pleiades Digital Elevation Model (DEM) in Global Mapper v15.0 software. Model shows different terrace levels; fault scarp (red line), trench (yellow rectangle).

**Figure 3:**
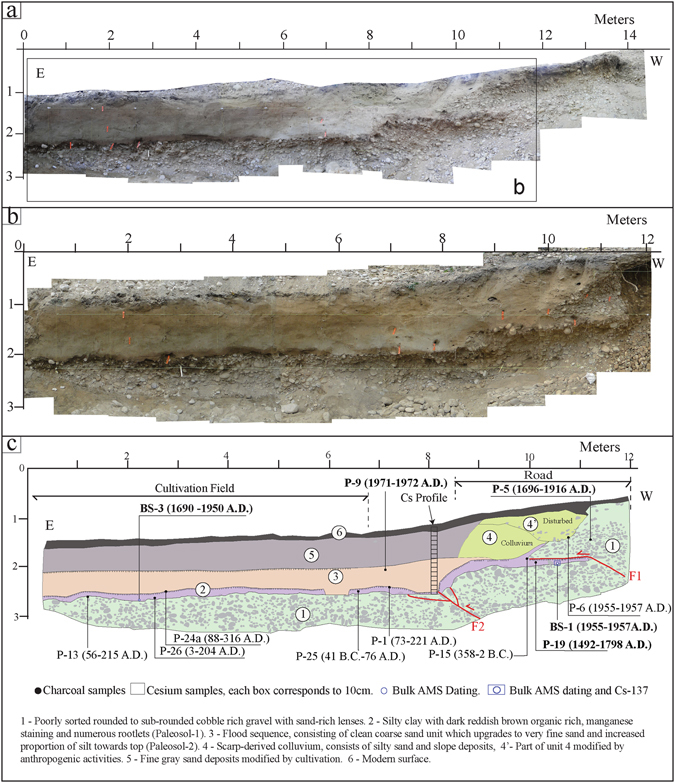
(**a**) Photomosaic of the excavated trench exposure at Pasighat. (**b**) Photomosaic of the trench exposure shown in Fig. 3c. (**c**) Illustrative log of the trench exposure shown in Fig. 3b showing different stratigraphic units in relation to the faulting event and the sample locations. Magnified image of the faulted portion of the trench exposure is provided in the Supplementary Fig. S1d. Mosaic of the trench photographs was done in Adobe Photoshop CS5 software and artwork in Adobe Illustrator CS5.

The study area Pasighat is located at the hinge zone of Siang antiform^24^, where the Quaternary landform comprises well preserved alluvial fans and terraces along the Siang (Brahmaputra) River and its tributaries (Fig. 2a,b,c and e). On the right bank of the Siang River, four levels of fluvial terraces (T1–T4) attest to the tectonic activity in the area. The terrace (T4) stands at a height of ~120 m, T3 at ~100 m, T2 at ~30 m and T1 at ~9 m relative to the current river grade (Fig. 2a). The lowest surface (T0) which stands at a height of ~6 m and stretches extensively is the floodplain of the Brahmaputra River. The T3 terrace is in actual a large fan surface emerging from the Siwaliks, that has been abruptly truncated and terminated at the front due to tectonic activity. The T1 and T2 terraces have been truncated by a NNE-SSW to NS striking tectonic scarp that gradually decreases in height from ~25 m in the southwestern end to ~3.1 m at the excavation site (~28.07°N, 95.33°E) of the trench (~14 m long and 4 m deep, see Figs 3 and S1a) in the northeastern end, and abruptly disappears with a ~E-W striking terrace riser (Fig. 2a and b). In the southern segment (Supplementary Figure S1e), the fault scarp striking NNW-SSE is more diffused due to modification by metalled road construction across the fault scarp and human settlement. Whereas in the northern segment, the fault scarp striking N-S is steep due to sharp transition from terrace T1 to T0 and less anthropogenic activities.

## Evidence of surface faulting at Pasighat

**Figure 4:**
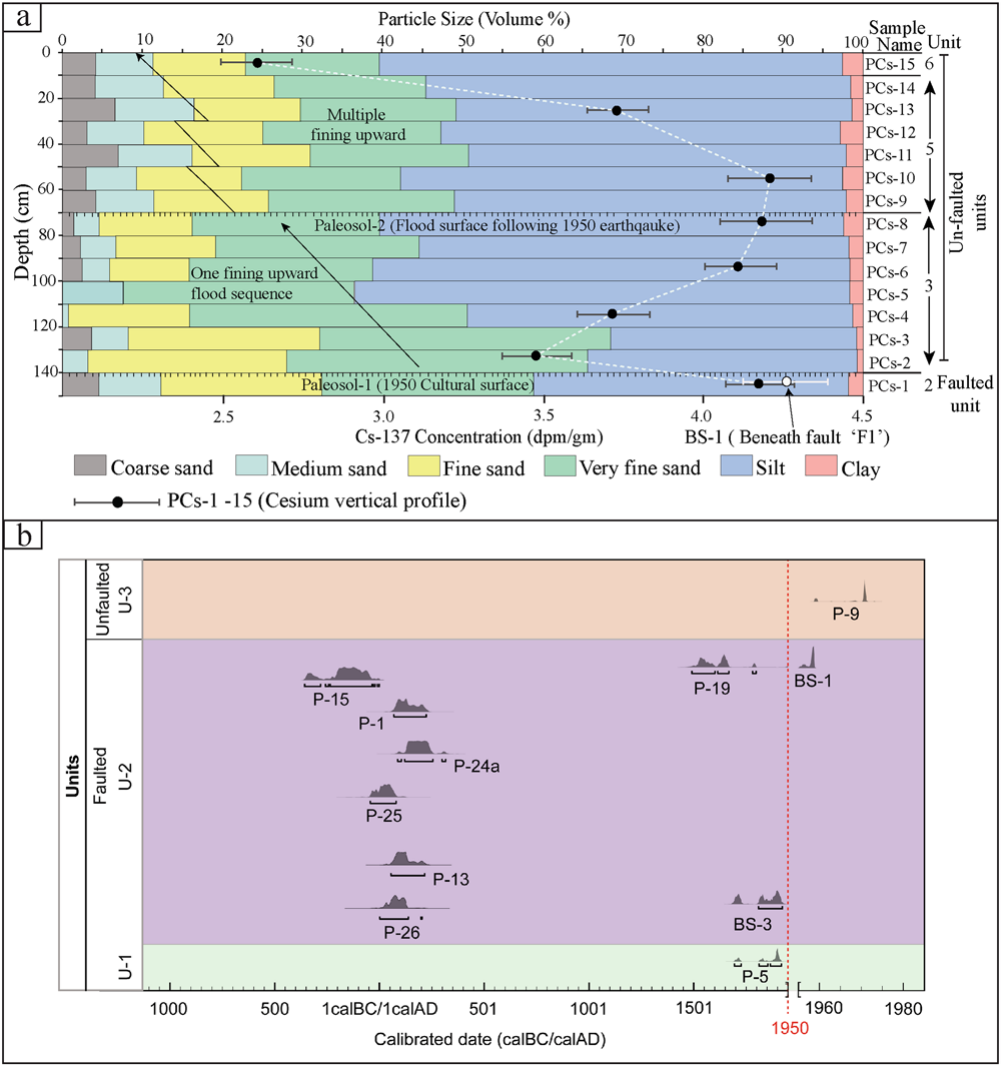
(**a**) Multi-variable vertical profile plot between grain size and ^137^Cs concentration as a function of depth (stratigraphy) in the Pasighat trench exposure. Black dots with lines on either side indicate the ^137^Cs concentration in the depth profile and its uncertainty. The sample BS-1 (white dot) from unit-2 palaeosol beneath fault ‘F1’ shows high Cs and ^210^Pb_xs_ concentration; high Cs concentration corresponds to the high clay content. (**b**) Probability Density Function (PDF) plot of radiocarbon samples obtained from the Pasighat trench exposure showing timing of the event. Plot generated in OxCal v4.3.1 online software.

The Pasighat trench exposures (Figs. 3 and S2b) were thoroughly studied and differentiated into different stratigraphic units from bottom through top on the basis of facies variation. The stratigraphy of the units is described in the trench log (Fig. 3c). The lowermost unit-1 consisting of poorly sorted rounded to sub-rounded cobble-rich gravels with sand-rich lenses, forms the base of the trench. The unit-2 (consisting of dark, organic rich silty clay that contains abundant charcoal with numerous rootlets) overlying unit-1 is interpreted as the palaeosol that formed prior to the faulting event which deformed it by two fault strands ‘F1’ and ‘F2’. The fault strand ‘F1’ that has placed the sediments of unit-1 over the palaeosol unit-2 has a dip of ~30–40° along the ramp which diminishes up-dip forming a flat. The fault strand F2 extends into the palaeosol unit-2 leading to the folding of the units-1 and 2 on the hanging wall. Minimum dip-slip displacement of ~1.4 is observed along the fault strand F1. Although the fault strand F2 does not show any significant displacement, it recorded ~0.5 m of vertical separation (Fig. 3c). Due to the deposition of a single colluvium package (unit-4) at the snout of the F1 strand, we interpret that both the fault strands F1 and F2 are coeval and were formed during the 1950 event. Soon after the surface faulting, a flood deposited the unit-3 which consists of sand with single fining-upward sequence (Fig. 4a) towards the top. Flooding deposited unit-3 on the footwall and effectively reduced the height of the fault scarp from 4.1 to 3.1 m. Progressively, the scraped off materials from the fault scarp, i.e., colluvium (unit-4) and the uniform fine sand unit-5 were deposited. The fine sand (unit-5) with multiple fining-upward sequence as indicated by grain size analysis (Fig. 4a) is interpreted to be sediments that deposited as flat lying stratigraphic unit along the scarp from periodic flooding of adjacent creeks and rivers. The units-4, 4′ and 5 were partly modified due to bioturbation and anthropogenic activities.

Ten detrital charcoal samples (P-1, P-5, P-6, P-9, P-13, P-15, P-19, P-24a, P-25 and P-26) collected from the southern trench wall were dated using ^14^C Accelerator Mass Spectrometer (AMS) technique, which range between 358 B.C. and A.D. 1972 (Figs. 3 and 4b). Five detrital charcoal fragments from the faulted and unfaulted units of the northern trench wall (P-3, P-7, P-18, P-22 and P-23) yielded calibrated ^14^C ages ranging between A.D. 88 and 1943 (Fig. S3). To overcome the shortcomings of detrital charcoal ages and to precisely constrain the timing of earthquake event, multiple proxies such as AMS radiocarbon dating of buried organic bulk soils (BS-1 and BS-3), radiocesium and *Total* ^*210*^ *Pb* (*Unsupported* ^*210*^ *Pb* + *Supported* ^*210*^ *Pb*) (10 samples) were employed (Supplementary Tables S1–S3). The organic bulk soil samples BS-1 and BS-3 were collected from palaeosol (unit-2) just beneath the fault strand ‘F1’ and ~5.5 m away from the F2 fault strand in the footwall, respectively. Loss On Ignition (L.O.I.) analysis of these bulk soil samples was also performed to check the reliability of the ages of bulk soils (Supplementary Table S2). Cesium (Cs) as well as ^210^Pb analyses of BS-1 were carried out along ﻿with﻿ a vertical Cesium (Cs) profiling (of 10 cm each) across the pre- and post-faulting units to determine the lateral as well as the vertical distribution of Cs (Figs. 4 and S2c and d and Supplementary Table S3). Radiocesium ^137^Cs isotope is produced as a global atmospheric fallout through atomic bombing and nuclear weapon testing and its presence provides a precise datum for sediments of age <75 years. The ^137^Cs isotopes can offer an important “first appearance” horizon of known age, providing much needed validation of the younger AMS ^14^C radiocarbon dating that may be associated with inbuilt age errors^25^.

**Figure 5:**
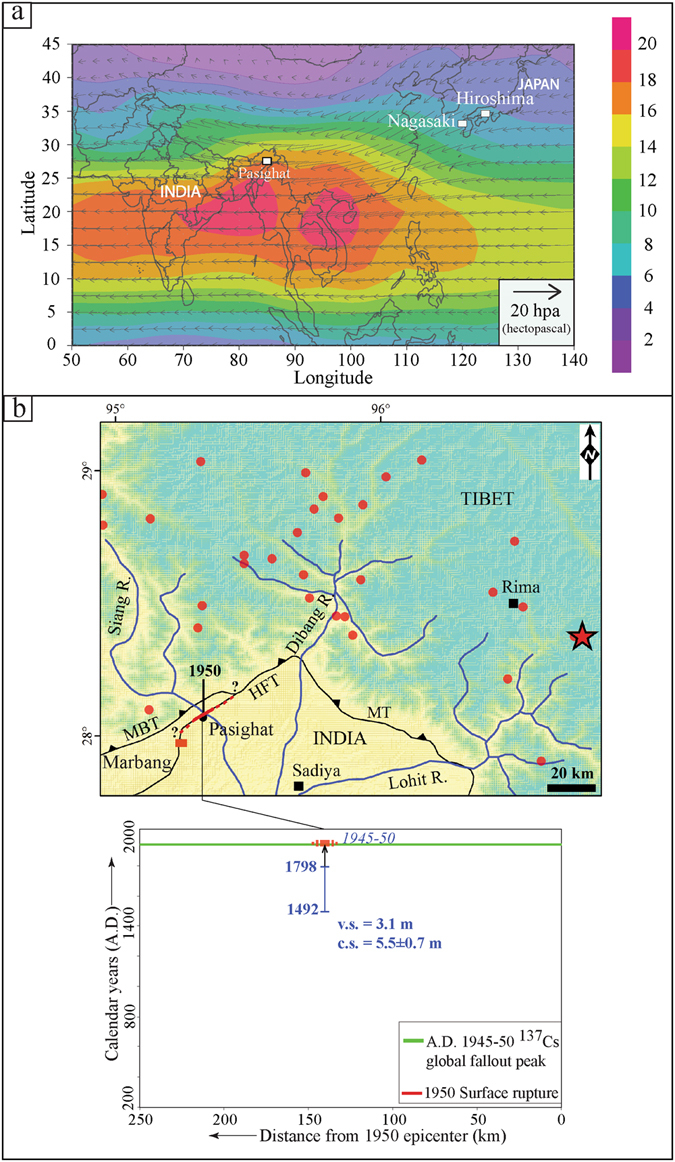
(**a**) Synoptic wind patterns (m/s) at 50-hpa during June 1948. Arrows indicate the wind direction; length of the arrow and color code correspond to the magnitude of the wind vector. Map was prepared in GrADS v2.0.2. software (Source: http://cola.gmu.edu/grads/). NCEP Reanalysis data provided by the NOAA/OAR/ESRL PSD, Boulder, Colorado, USA, from their website at http://www.esrl.noaa.gov/psd (**b**) **Top**: Map showing the discovered earthquake surface rupture of 1950 at Pasighat and dashed line denotes uncertainty; 1950 epicenter from a previous study^15^ (red star); aftershocks (red dots); major thrusts (HFT-Himalayan Frontal Thrust, MBT-Main Boundary Thrust, MT-Mishmi Thrust). Map was prepared in Arc GIS v9.3 using SRTM GTOPO 30 m imagery available at http://glcfapp.glcf.umd.edu/data/srtm/description.shtml. **Bottom:** Figure showing the spatiotemporal distribution of 1950 rupture event observed in the Pasighat trench (red solid line) and the uncertainty (dotted); v.s. = vertical separation; c.s. = co-seismic slip. Ages in italics denote Cs peak. Figure was designed in Adobe Illustrator CS5 software.

To examine whether the concentration of Cs is *in-situ*, we performed grain size analysis (Fig. 4a) along with clay mineralogy (Supplementary Fig. S4b and Table S4) to check the possibility of vertical downward migration as well as its physical and mechanical mixing by bioturbation. The above mentioned analyses suggested the following. If detrital charcoal sample P-19 (ranging from A.D. 1492–1798) collected just beneath the fault strand ‘F1’ was *in-situ*, then the earthquake might have occurred after A.D. 1492–1798 (Fig. 4b). The bulk soil ages ranging from A.D. 1690–1957 (modern age) may be ascribed to the carbon budget error associated with bulk organic sediments related to continuous supply of rain water^26^. However, high values of total organic content (T.O.C.) for the BS-3 (A.D. 1692–1950) sample negate this and suggest that the unit-2 (palaeosol) existed as a surface during 1950 (Supplementary Table S2). Cesium analysis of the sample BS-1 collected from beneath the fault strand ‘F1’ along with the sample PCs-1 (Supplementary Table S3) from the faulted unit-2 palaeosol reveals significantly higher concentration of radiocesium isotope. This implies that the unit-2 was exposed to receive the radiocesium fallout isotopes from the atmosphere prior to the surface faulting along fault F1 (Supplementary Figs. 4a, S2c and d). The Cesium concentration in the unit-2 corroborates the first transcontinental transport of ^137^Cs fallout isotope from Hiroshima-Nagasaki (Japan) atomic bombing to the Indian subcontinent. Wind patterns of 1948 (earliest available data) analysis of global fallout for different altitudes in the Troposphere and Stratosphere suggest that the wind direction at 50 hPa (20 km) during July was favorable for the transportation of ^137^Cs (half-life ~30 years) from Nagasaki to India (Fig. 5a). Residence time of the particles and gases in the Stratosphere is a few years before their dispersal to other regions and/or lower altitudes as global fallout. Presence of ^137^Cs in the Arctic ice core layer of Canada and global fallout of ^137^C suggest that post Nagasaki explosion ^137^C was transported globally, owing to its high ‘Transport Ratio’ (67%)^27^.

Generally, due to radioactive decay the concentration of ^210^Pb_xs_ decreases with depth in a stable environment of normal stratigraphic sequence^28, 29^. However, the samples PCs-2 to PCs-14 (Units-4 and 5, Supplementary Table S3) show no ^210^Pb_xs_ because of the rapid deposition of these units, which accords with a suggested flood event soon after the earthquake^30^. Contrastingly, presence of unsupported ^210^Pb (i.e., ^210^Pb_xs_) in BS-1 at a depth of ~2 m (Supplementary Table S3) indicate stabilization of the palaeosurface and sub-aerial exposure of the unit prior to the faulting. Granulometric analysis and clay mineralogy suggest that ^137^Cs did not migrate downward by physical and/or mechanical processes, and its vertical migration, if any, was further blocked due to the presence of expandable clay minerals^31, 32^ (Fig. 4a and Supplementary Table S4). In general, the mobility of ^137^Cs is low in clay and organic rich soil, and clay mineralogy is the most important factor favoring radiocesium immobilization^31, 32^. Therefore, all these evidences suggest that the unit-2 was the cultural layer at the time of 1950 earthquake. Unit-1 yielded two charcoal samples P-5 and P-6. The sample P-6 is from a disturbed zone incorporated with polythene bags, randomly oriented gravels are also seen in the opposite wall. Hence the sample P-6 was not considered for further interpretations. Antiquity of sample P-5 ranges between A.D. 1696 and 1916, a minimum age of unit-1.

## Discussions and Conclusion

Our study signifies that the 1950 Tibet-Assam earthquake, the highest recorded magnitude (*M*_*w*_ ~ 8.6) in the Himalaya^33, 34^ did break the Himalayan front. Considering a dip of 55–60° for the causative fault^15, 16^, we interpret that the scarp of ~3.1 m height at Pasighat has been produced due to a co-seismic slip of ~5.5 ± 0.7 m during the 1950 earthquake (Supplementary Table S5). If we consider fault dip from the trench exposure with interpreted vertical separation of ~4.1 m, then the co-seismic slip would be ~7.3 ± 0.9 m (Supplementary Table S5). Estimated average slips for the causative fault was 16 m for a low angle thrust fault^15^ and 6.6 m for a strike slip fault^16^ on a rupture area of 250 × 80 km^2^ considered in both the cases. However, a minimum of ~1.4 m slip on fault strand F1 is insufficient to account for the vertical separation (4.1 m) across the fault. The incongruity between slip and scarp height might be due to factors such as change in geometry of the thrust ramp beneath the scarp, insufficient depth of the excavated trench to expose the piercing points of the marker horizons across the fault, near surface folding of surficial layers as well as folding and faulting of the units outside the visibility of the trench exposure, and tilting of the stratigraphic units^3, 13^. Interestingly, the height of the scarp reported in this study is very low as compared to other trenches excavated elsewhere along the Himalayan arc^1, 3, 9^. Further, strike of the trenched fault scarp is NNW-SSE to NS which is in agreement with one of the nodal plane for the strike-slip fault mechanism^16^. Considering a smaller height of the scarp with ~NS strike and evidence from the post-earthquake damage photographs taken by the Geological Survey of India (GSI) (Supplementary Fig. S7), it may be suggested that the surface faulting was characterized in the form of oblique thrust fault mechanism which may be due to the structural complexity arising from the juxtaposition of two structural grains of the Himalaya, i.e., ~ENE-WSW for the HFT and NW-SE for the MT.

Evidences of contemporaneous damages that occurred extensively in and around the Pasighat town (reported by eye-witnesses) were collectively published in the Central Board of Geophysics report of 1953 by M. B. Ramachandra Rao (Supplementary Fig. S7). The 1953 report mentions that the Kobo-Pasighat road between the mile 6 and 14 was damaged by the earthquake and the subsequent flood^30^. It has also been mentioned that, the Lohit and Dibang river beds subsequently uplifted by several meters. A dam on the Brahmaputra River to the north of the Pasighat town broke, causing serious flooding. This might be the reason for the inaccessibility of the area subsequent to the earthquake, due to which the Pasighat town could not be examined by the GSI workers and thus the scarp remained unnoticed^35^. All these evidences collectively suggest that the seismic event recorded in the Pasighat trench corresponds to the A.D. 1950 Tibet-Assam earthquake.

Our study thus confirms that the 1950 earthquake ruptured the Himalayan front at longitude ~95° 33′E (Fig. 5b). Our interpretation of an oblique thrust fault mechanism for the causative fault of the 1950 earthquake is in agreement with the GPS derived fault plane solution for the earthquake^36^. For fault dip angle of 35 ± 5° obtained from the trench and scarp height of 4.1 m, an oblique slip of 10.3 ± 1.3 m is obtained. However, considering a dip angle of 55° for the causative fault^16^ and scarp height of 4.1 m, an oblique slip of 6.9 ± 0.2 m is obtained (Supplementary Table S5). In both the cases, the angle of obliquity assumed is 45° as per a previous GPS study^36^. This paper provides the first evidence of transcontinental transport of radioactivity related to the Hiroshima-Nagasaki atomic bombing (1945) to the Indian subcontinent.

## Methods

### High resolution imagery analysis and micro-topographic surveying

For the identification of fault scarps and disjointed geomorphic surfaces along the active Himalayan front, Pleiades and Cartosat-1 satellite imageries were used in SOCKET-GXP software. These geomorphic features were further examined in the field by conducting high resolution aerial survey using Phantom cod copter or Unmanned Aerial Vehicle (discussed in the subsequent section) (Fig. 2c) to identify the youngest fault scarps and their lateral extent for the purpose of palaeoseismological investigations (Supplementary Fig. S1).

Real Time Kinematic Global Positioning System (RTK-GPS) and Robotic Total-Station were used to survey the geomorphic features at each site (Supplementary Fig. S1e and f), and measuring topographic profiles across the fault scarps offsetting the youngest geomorphic surfaces (Figs 2a,b,c,e and S1f). The RTK-GPS kinematic data were processed using Leica Geo Office (LGO) software.

### Topographic Structure-from-Motion (SfM)

High resolution Digital Elevation Models (DEM) of the area around the trench site, developed from Structure-from-Motion (SfM) processing of aerial photographs acquired from Unmanned Aerial Vehicle (UAV) provided analytically accurate information about geomorphic landscape and geological outcrop^37–40^ (Fig. 2c and d).

During flight, photographs were captured along the fault trace every 5 seconds at a lower altitude of <30 m, using Ricoh GR 8.3 megapixel digital camera mounted at the bottom of DJI Phantom 1 multi-rotor cod copter. Thus, based upon the area of the site ~120–500 photographs were acquired at each site. Prior to the flight, ground control points (GCP) arrayed at 5 to 20 meters apart, depending upon the area traversed during the flight, were established to constrain the scale in the modelling process. All the points were geospatially located using Real Time Kinematic Global Positioning System (RTK-GPS) with an accuracy of ±5 mm. All acquired images went through photogrammetric processing that included interior and exterior orientation, using open source software package AgiSoft Photo Scan Pro. A dense cloud of the photographs was created using the default settings in the software and a Triangular Irregular Network (TIN) was generated. DEM were generated using ArcGIS software (Fig. 2c).

### ^14^C dating

To obtain a ^14^C age for the timing of earthquake event/s, 17 charcoal samples (12 samples from the southern and 5 from the northern wall) collected from different faulted and unfaulted units exposed in the Pasighat trench were dated (Figs 3c and S3b and Table S1). The detrital charcoal fragments and bulk soil samples were analyzed at the Poznań Radiocarbon Laboratory (Poland) and Beta Analytic (Florida) by Accelerator Mass Spectrometry. All the reported radiocarbon dates are of 2σ (95% confidence limits) calendar age ranges in B.C. or A.D. calibrated with CALIB program v7.0.4^41^.

The ages of samples should decrease in some regular manner as one progress stratigraphically upward^1, 4, 14, 25, 42^. An older age sample outside of the progression is a clear indication of reworked charcoal which gives an age older than the horizon in which it is preserved (Figs 3c and S3b). Probability Density Function (PDF) plot of radiocarbon samples obtained from the Pasighat trench exposure showing timing of the event was generated in OxCal v4.3.1 online software.

Our concern for ^14^C dating of bulk soil from unit-2 palaeosol of Pasighat trench is that, the continuous supply of organic matter to a palaeosurface can give either younger or older ages, therefore, the total organic content (T.O.C.) was determined (Supplementary Table S2) for the accurate interpretation of the event age for the unit from which the sample was obtained^43^.

### Total Organic Content (T.O.C.) estimation

The organic sediments in a palaeosurface reveal complex carbon budget where the radiocarbon ages of the different constituents do not match with the actual time of sedimentation. It is well known that the ^14^C age of bulk organic sediment samples is affected by a number of error sources^44^. However, it has long been recognized that the abundant organic content of sediments improve results considerably, since they are prone to record disturbances such as hard water effect or mechanical contamination. Therefore, relatively high organic content and insufficient carbonate content in soil horizon excludes reservoir effect^45^. To rule out the possibility of the error associated with AMS dating of bulk organic sediments, we analyzed the sediment properties such as organic matter, moisture and impurity content of unit-2 palaeosol. Loss-On-Ignition (L.O.I.) analysis was carried out for bulk soil samples BS-1 and BS-3 from the same unit (Fig. 3c and Supplementary Table S2).

In the Pasighat trench, the total organic content was measured as per standard procedure^45, 46^ at 550 °C on samples BS-1 and BS-3 are 2.63% and 3.32% (Supplementary Table S2). Significant measures of the organic matter content of the two samples clearly indicate the *in-situ* existence of the unit from which the samples were analyzed, thus supporting the consistency of the AMS radiocarbon ages. The AMS ages of the bulk organic samples (BS-1 ranging from A.D. 1955–1957 and BS-3 from A.D. 1690–1950) obtained from unit-2 palaeosol are younger than the detrital charcoal P-19 (A.D. 1492–1798) displaying a small age difference, though collected at the same level from the same unit (Figs. 3c and S2c). Moreover, the detrital charcoal fragment P-9, found in the overlying un-faulted unit-3 yielded an age A.D. 1971–1972, younger than the bulk sediment ages suggesting the most recent event took place prior to A.D. 1971–1972 (Figs. 3c and S2c). The timing of the most recent event found in the trench is further constrained by the concentration of ^137^Cs global fallout isotopes.

### Analyses of ^137^Cs and ^210^Pb global fallout radioactive isotopes

Wide use of nuclear weapons as atomic bombing during the year 1945 and their testing till 1975 resulted in the exhaustion of fallout isotopes such as ^137^Cs, ^210^Pb and ^239+240^Pu. These isotopes are widely used as chronomarkers in sediments for construction of high resolution chronologies of age <75 years^27, 47^.

The isotopic concentrations of ^210^Pb, ^137^Cs and ^226^Ra were determined by non-destructive gamma counting method^48^. Analytical precision of this technique is ±5% (1σ) based on multiple analyses of several standards used for calibration^49–51^.

Considering that the radioactive fallout from the first USSR test (1949), atomic explosion in New Mexico, USA (1945), and atomic bombing at Hiroshima and Nagasaki, Japan (August 6 and 9, 1945) was deposited more than 50 years ago, other than physical decay of radionuclides, the current distribution of radioactive contamination is reflecting various factors such as migration into soil, washing out, re-suspension, human activities and so on ref. 27. Prior studies demonstrated first noticeable appearance of ^137^Cs during 1945–50, thus the peak which appeared first is considered as 1950 peak^27^. Some other peaks of ^137^Cs were observed during 1963, 1966 (nuclear), 1986 (Chernobyl), 1998 (India), 2011 (Fukushima)^28, 52^.

Total ^210^Pb in a gradually depositing environment is made of two components. Firstly, supported ^210^Pb component is derived from the radioactive decay of parent ^226^Ra which is believed to be in secular radioactive equilibrium and equal to parent ^226^Ra concentration. Secondly, unsupported ^210^Pb component (also called^210^Pb_xs_) is derived from atmospheric fallout due to the escape of ^222^Rn from the U-series radionuclide which decays rapidly to ^210^Pb and deposited by rain and/or dry fallout. The total ^210^Pb was measured as follows:

$${}^{{210}}Pb(Total)={}^{{210}}Pb(Supported)+{}^{{210}}Pb(Un{supported})$$

The concentration of ^210^Pb_xs_ is high at the surface and decreases with depth, as a result of radioactivity decay^29, 53^. The ^210^Pb_xs_, which decreases with the half-life of ~22.5 years helps in determination of sedimentation rate. The fallout radionuclide is rapidly and strongly adsorbed by the surface soil, unsupported ^210^Pb behaves in a similar manner as ^137^Cs^54^.

The prepared samples were analyzed for ^137^Cs and ^210^Pb concentrations at Physical Research Laboratory, India.

### Application of radioactive isotopic dating in the Pasighat trench

A vertical profile of 150 cm comprising 15 sediment samples (PCs-1 to 15) was collected from adjacent segments of 10 cm each from the southern wall of the Pasighat trench (Supplementary Fig. S2c and d). Of these samples, we analyzed 9 samples (PCs-1, 2, 3, 4, 6, 8, 10, 13 and 15) from the faulted unit-2 palaeosol, and the un-faulted units-3, 4 and 5 along with BS-1 sample from unit-2 collected beneath the fault strand ‘F1’ (Figs 3c and S2c) in anticipation of possible concentrations of ^137^Cs and ^210^Pb (Fig. 4a and Supplementary Table S3).

The measurements of ^210^Pb in Pasighat trench show distorted and scattered activities. In regard to the samples PCs-2 to PCs-14 shows no ^210^Pb_xs_. The measurement of ^210^Pb in BS-1 from unit-2 palaeosol shows high concentration of ^210^Pb_xs_ (Supplementary Table S3).

In case of ^137^Cs, peaks of ^137^Cs have been recorded in the units-2 (palaeosol), 3 and 5, reaching 4.12 DPM gram^−1^, 4.23 DPM gram^−1^, 4.20 DPM gram^−1^ and 4.21 DPM gram^−1^, for samples PCs-1, BS-1, PCs-8 and PCs-10, respectively (Fig. 4a and Supplementary Fig. 2c). The upper part of unit-3 is characterized by buried soil referred to as Palaeosol-2 that shows high concentration of Cs, but it progressively decreases with depth and again reaches higher concentration at lower buried soil referred to as Palaeosol-1 of unit-2.

Sample BS-1 shows higher concentration of both ^137^Cs and unsupported ^210^Pb (Fig. 4a and Supplementary Table S3), indicating prolonged aerial exposure of the surface for the deposition of atmospherically fallout radionuclide prior to the faulting event recorded in the trench exposure. We report an appreciable ^137^Cs concentration in unit-3, but no ^210^Pb_xs_ observed, which thus indicates the rapid deposition of the sediments as in a flood event associated with the 1950 earthquake^30^. Thus, unit-3 is the capping unit post-dating the earthquake event. Since no decipherable ^210^Pb_xs_ is observed with depth due to rapid sedimentation, the calculation for sedimentation rate is not possible.

In the trench exposure, no signatures of bioturbation were observed in units-2 and 3, whereas, units-4, 4′ and 5 have been affected by bioturbation and anthropogenic activities (e.g., plantation, cultivation and unmetalled road construction). In order to confirm whether the concentration of ^137^Cs in the unit-2 palaeosol is *in-situ* and has not been mobilized by vertical migration through overlying units due to rainwater percolations or by the physical mixing due to bioturbation, grain size analysis was further carried out along with clay mineralogy (Fig. 4a). In general, the mobility of ^137^Cs is low in clay and organic rich soil, and clay mineralogy is the most important factor favoring radiocesium immobilization^31, 32^. The low mobility of ^137^Cs is mainly due to the specific sorption and fixation of ^137^Cs by the presence of expandable clay minerals such as Vermiculite, Smectite and Illite (Supplementary Table S4; Discussed in the subsequent section).

### Grain Size Analysis

For the determination of vertical ^137^Cs distribution, grain size analysis along with quantification of clay mineralogy were undertaken for the same profile that was used for the measurement of radiocesium in the Pasighat trench (Figs 4a and S2C). In fact, mobility of ^137^Cs is hindered in sediments with high percent of clay content^32, 53, 55–57^. The grain size analysis was performed by Laser Particle Size Analyzer (LPSA) Mastersizer 2000, having sensitivity of <1 mm using standard procedures^58, 59^ at Wadia Institute of Himalayan Geology, Dehradun.

Analysis of grain size indicates a single fining-upward sequence in the unit-3 (Fig. 4a), which very well correlates with the flood event subsequent to the 1950 earthquake^30^. Multiple flux of coarse and medium sand along with fluctuations in the silt and clay fraction in unit-5 show multiple fining-upward sequences suggesting that this unit was deposited as growth stratigraphy by adjoining creeks during the monsoon. Subsequently, this unit was disturbed by anthropogenic and/or bioturbation activities.

In the Pasighat trench, unit-2 palaeosol shows significant percent of clay to hold a higher concentration of ^137^Cs radionuclide and further check its mobility (Fig. 4a and Supplementary Table S4). In unit-3, the samples PCs-8 and 10 show high percentage of clay content which recorded higher concentration of Cs. Besides the clay content, clay mineralogy (Fig. 4a and Supplementary Table S4) seems to be the important factors favoring radiocesium immobilisation^32, 53, 55–57^.

### Clay Mineralogy

From the sample prepared for the grain size analysis, 5 g of sub-sample is extracted from each sample to identify the type of clay mineral present in the sample (Supplementary Table S4) using standard laboratory protocols at Wadia Institute of Himalayan Geology, India.

### Qualitative identification of clay minerals

The clay minerals were identified on the basis of their respective peaks of different clay minerals and their distinct value of d-spacing. For the identified clay minerals present in our analysis, there were schematic variations in their respective peaks^60^ (Supplementary Fig. S4b and Table S4). The technique of semi-quantitative estimation utilizes the height of specific reflections measured in general in the Ethylene-Glycol (EG) runs. The semi-quantitative classification of the clay minerals was based on Biscaye method^61^ and the area of their respective peaks was computed with EG.

Analysis of samples PCs-1 to 15 obtained from the Pasighat trench exposure indicates appreciable presence of clay and flaky minerals, Illite and Chlorite (Supplementary Fig. S4b and Table S4]. High percentage of Illite for samples PCs-9 to14 (unit-5), PCs-15 (unit-6) and PCs-8 and 6 (unit-3) blocks the downward migration of the ^137^Cs radionuclide because of high adsorption of radiocesium on Illite particles (the frayed edge of Illite)^31^. The grain size analysis along with quantification of clay mineralogy show no migration of radiocesium.

In summary, the depth distribution of ^137^Cs in the soil or sediments can only be partly explained by different physicochemical soil properties and bioturbation as mentioned in the preceding paragraphs. Thus other factors such as rainfall intensity, infiltration rates and water flow in the soil also influence the depth distribution of radiocesium. However, higher concentration of ^137^Cs in the clay-rich unit suggests that the ^137^Cs concentration found in the unit-2 palaeosol of Pasighat trench (Fig. 4a) is *in-situ* and this unit is the event horizon of the 1950 earthquake, which was exposed to atmosphere to receive the fallout origin of radiocesium pertain to pre-1950 atomic testing and bombing.

### Wind Modelling

In 1945, two atom bombs were successively dropped at the twin cities of Hiroshima and Nagasaki in Japan at a height of ~503 m. The altitude of the atomic cloud reached into the stratosphere at 8–10 km for the global fallout^27^ (Fig. 5a).

The radioactive cloud usually takes the form of a mushroom, that familiar icon of the nuclear age. As the cloud reaches its stabilization height, it moves downwind, and dispersion causes vertical and lateral cloud movement. Because wind speeds and directions vary with altitude, radioactive materials spread over large areas. Large particles settle locally, whereas small particles and gases may get transported to far-off places.

Wind directions at 2.5° latitude and longitude resolution at 50 hPa speed for June 1948 are drawn using National Center for Environmental Prediction (NCEP) Reanalysis data (Fig. 5a). NCEP Reanalysis provides a four-dimensional gridded data of wind direction and wind speed as a function of latitude, longitude, time and height. NCEP reanalysis of wind velocity available at 17 levels between 1000 and 10 hPa is an ‘A-type’ field which is strongly influenced by the observed data and is most reliable, available from 1948. Since, data for 1945 was not available we have used data corresponding to 1948, this choice is justified as we are tracing particles and gases that reside in the stratosphere for 3 to 5 years.

Our wind analysis of 1948 suggests that radioactive clouds were transported by strong easterly winds to northeastern India (Fig. 5a), where fallout on the sediments occurred by a process of dry deposition of aerosols. The results of the present work confirms for the first time, evidence of the Nagasaki-Hiroshima fallout isotope in the Indian subcontinent.

## Electronic supplementary material

Supplementary Information
