# Supplementary material for: Primary surface rupture of the 1950 Tibet-Assam great earthquake along the eastern Himalayan front, India
Source: Sci Rep. 2017 Jul 14;7:5433. doi: 10.1038/s41598-017-05644-y (PMC5511192; doi:10.1038/s41598-017-05644-y)
Supplement: Supplementary file 1 — Supplementary Information [file 41598_2017_5644_MOESM1_ESM.pdf]

Primary surface rupture of the 1950 Tibet-Assam great earthquake along the eastern Himalayan front, India

Rao Singh Priyanka[1,2], R. Jayangondaperumal[1]*, Arjun Pandey[1], Rajeeb Lochan Mishra[1], Ishwar Singh[1], Ravi Bhushan[3], Pradeep Srivastava[1], S. Ramachandran[3], Chinmay Shah[3], Sumita Kedia[4], Arun Kumar Sharma[5], Gulam Rasool Bhat[6]

[1]Wadia Institute of Himalayan Geology, Dehradun, India

[2]Geological Survey of India, SU: WB & AN, ER, Kolkata, India.

[3]Physical Research Laboratory, Ahmedabad, India

[4]Centre for Development of Advanced Computing, Pune, India

[5]Department of Geology, Kumaun University, Nainital, India.

[6]Department of Geology, Pondicherry University, Port Blair Campus, India.

*Correspondence to: ramperu.jayan@wihg.res.in , ramperu.jayan @gmail.com

Tables

Table S1. AMS Radiocarbon (^{14}C) dates.

Table S2. L.O.I analysis for Bulk Soil samples.

Table S3. Total ^{210}Pb and ^{137}Cs concentrations.

Table S4. Clay mineral composition of different stratigraphic units.

Table S5. Estimated fault slip.

Figures

Figure S1. Field photograph of scarp and trench site; photo logs of southern and northern walls; micro-topographic map of the trench site and scarp profile.

Figure S2**.** Photographic log of the southern trench wall; enlarged view of fault zones and Cesium profile in the southern trench wall; photo showing the Cesium sampling from the trench exposure.

Figure S3. Photographic and illustrative logs of the northern wall of Pasighat Trench.

Figure S4. Depth plot of the radiocarbon samples and X-ray diffraction diagrams.

Figure S5. Probability Density Function (PDF) plots of two modern samples obtained from the Pasighat trench.

Figure S6. Progressive stages of deformation by the 1950 earthquake.

Figure S7. Excerption from post-earthquake damage report of the Geological Survey of India.

Unit[a]	Sample Id	Lab Code[b]	Uncalibrated Conventional Radiocarbon Age[c]	$\partial^{13}C$ Reported[d]	Calibrated Ages (Calendric,2σ)[e]
Southern Wall, Pasighat Trench					
1	P5	Poz#2-73565	30 ± 30 BP	-37.4	A.D. 1696-1916
2	P19	Poz#2-71089	275 ± 35 BP	-43.2	A.D. 1492-1798
2	P1	Poz#2-73494	1875 ± 30 BP	-23.8	A.D. 73-221
2	P15	Poz#2-73504	2120 ± 50 BP	-12.2	B.C. 358-2 B.C.
2	P13	Poz#2-73563	1890 ± 30 BP	-39.4	A.D. 56-215
2	P24a	Poz#2-73569	1825 ± 30 BP	-42.6	A.D. 88-316
2	P25	Poz#2-73570	1975 ± 30 BP	-19.9	B.C. 41-A.D. 76
2	P26	Poz#2-73571	1920 ± 30 BP	-20.8	A.D. 3-204
2	BS-1	Poz#2-73579	103.2 ± 0.3 pmc	-24.1	A.D. 1955-1957*
2	BS-3	Poz#2-73581	70 ± 30 BP	-26	A.D. 1690-1950
3	P9	Poz#2-71087	148.83 ± 0.44 pmc	-34.1	A.D. 1971-1972*
4'/1	P6	Poz#2-71086	102.85 ± 0.33 pmc	-32	A.D. 1955-1957*
Northern Wall, Pasighat Trench					
2	P-23	Poz#2-73568	1825 ± 30 BP	-32	A.D. 88-317
3	P-7	Poz#2-73564	135 ± 30 BP	-36.6	A.D. 1671-1943
3	P-22	Poz#2-73567	1760 ± 30 BP	-16.6	A.D. 171-383
4	P-18	Poz#2-71088	1750 ± 30 BP	-21.1	A.D. 222-385
5	P-3	Poz#2-71085	1775 ± 30 BP	-17.4	A.D. 138-339

[a]Refer respective trench logs for stratigraphic units designations.

[b]Samples have been analysed at Poznań Radiocarbon Laboratory (Poland) and Beta Analytic (Florida) by accelerator mass spectrometry (AMS). Each number corresponds to the laboratory code for each sample.

[c]Conventional Radiocarbon years B.P. relative to 1950 A.D. with 1σ confidence level comprising of counting statistics, reference standard, blank and random machine error.

[d] The $\delta^{13}C$ values are the assumed values according to Stuvier et al. (1998)when given without decimal places. Values measured for the material itself are given with a single decimal place.

[e]Pre-bomb calendric dates were calibrated using CALIB v7.0.4[41].

*Post bomb samples calibrated using CALIBomb.

Table S1. AMS Radiocarbon (^{14}C) dates from detrital charcoals collected from Pasighat trench. All samples are detrital charcoals except BS1 and BS3, which are buried organic soils.

Sample	Unit	L.O.I. at (wt%)					^{14}C age (in A.D.)
		110˚C	550˚C (Organic)	950˚C (Inorganic)	110˚C-550˚C (Organic)	550˚C-950˚C (Inorganic)	
BS-1	2	6.70	9.33	10.01	2.63	0.67	1955-1957
BS-3	2	11.44	14.76	15.48	3.32	0.72	1690-1950

Table S2. L.O.I analysis for Bulk Soil samples derived from faulted unit-2 palaeosol of the Pasighat trench.

Sample Id	Depth from top surface (cm)	Unit Number	Pb-210 (Total)		Cs-137		Pb-210 (excess)
			DPM/g	Error	DPM/g	Error	
Pasighat Trench							
PCs-1	140	2	1.87	0.39	4.12	0.11	-
PCs-2	130	3	2.35	0.40	3.48	0.11	-
PCs-3	120	3	-	-	-	-	-
PCs-4	110	3	2.91	0.42	3.73	0.12	-
PCs-6	90	3	2.34	0.48	4.12	0.13	-
PCs-8	70	3	1.65	0.54	4.20	0.15	-
PCs-10	50	5	2.43	0.45	4.21	0.13	-
PCs-13	20	5	1.81	0.34	3.74	0.10	-
PCs-15	0	6	0.24	0.54	2.61	0.14	-
BS-1	140	2	3.50	0.10	4.27	0.45	2.36

Table S3. Total Pb-210 and Cs-137 concentrations along with Pb-210 excess for the samples collected from the southern wall of the Pasighat trench. Here DPM/g stands for "Disintegration per minute per gram" of the sample and BDL stands for "Below Detection Limit".

Sample Id	Unit Number	Depth (cm)	Illite	Chlorite	Kaolinite
PCs-1	2	140	++	+++	+++
PCs-2	3	130	++	+++	+++
PCs-3	3	120	+++	++	++
PCs-4	3	110	+++	++	++
PCs-5	3	100	+++	+++	+
PCs-6	3	90	+++	+++	++
PCs-7	3	80	++	+++	++
PCs-8	3	70	+++	++	++
PCs-9	5	60	+++	+++	++
PCs-10	5	50	++	+++	+++
PCs-11	5	40	+++	+++	++
PCs-12	5	30	+++	+++	++
PCs-13	5	20	+++	+++	++
PCs-14	5	10	+++	+++	++
PCs-15	6	0	+++	+++	++

+++ = Highest intensity (>30%)
++ = Moderate intensity (20-30%)
+ = Low intensity (<20%)

Table S4. Clay mineral composition of different stratigraphic units of the Pasighat trench.

Scarp height[$] (m)	Fault Dip (in degree)		Coseismic dip slip[@] (m) (A)	Coseismic dip slip[@] (m) (B)	Coseismic Oblique slip[¥] (m) (A)	Coseismic Oblique slip[¥] (m) (B)
	**Trench exposure* (Θ) (A)**	**Inferred instrumental [#] (Θ) (B)**				
3.1	30-40	55-60	5.5±0.7	3.7±0.1	7.8±1.0	5.2±0.1
4.1	30-40	55-60	7.3±0.9	4.9±0.1	10.3±1.3	6.9±0.2

$ denotes scarp height obtained from the RTK-GPS is shown in Fig. 2d. Scarp height 3.1 m (when measured as the vertical difference between the sloping profile across the scarp) and scarp height 4.1 m (when measured as the vertical separation between the sloping profile of the hangingwall and the event horizon unit-2 in the footwall)

@ denotes the coseismic dip s$lip\ (m) = \frac{Scarp\ height\ (m)}{sin\theta}$, where, θ represents the dip of the fault

¥ denotes the coseismic oblique slip (m) = amount of dip slip / cos α**,** where α is the angle of obliquity (45^0) derived from the angle between GPS vector and structural trend of HFT[36]

# denotes the instrumental dip referred from previous study[15,16]

* denotes the measured dip in this study.

Table S5. Estimated fault slip in the present study.

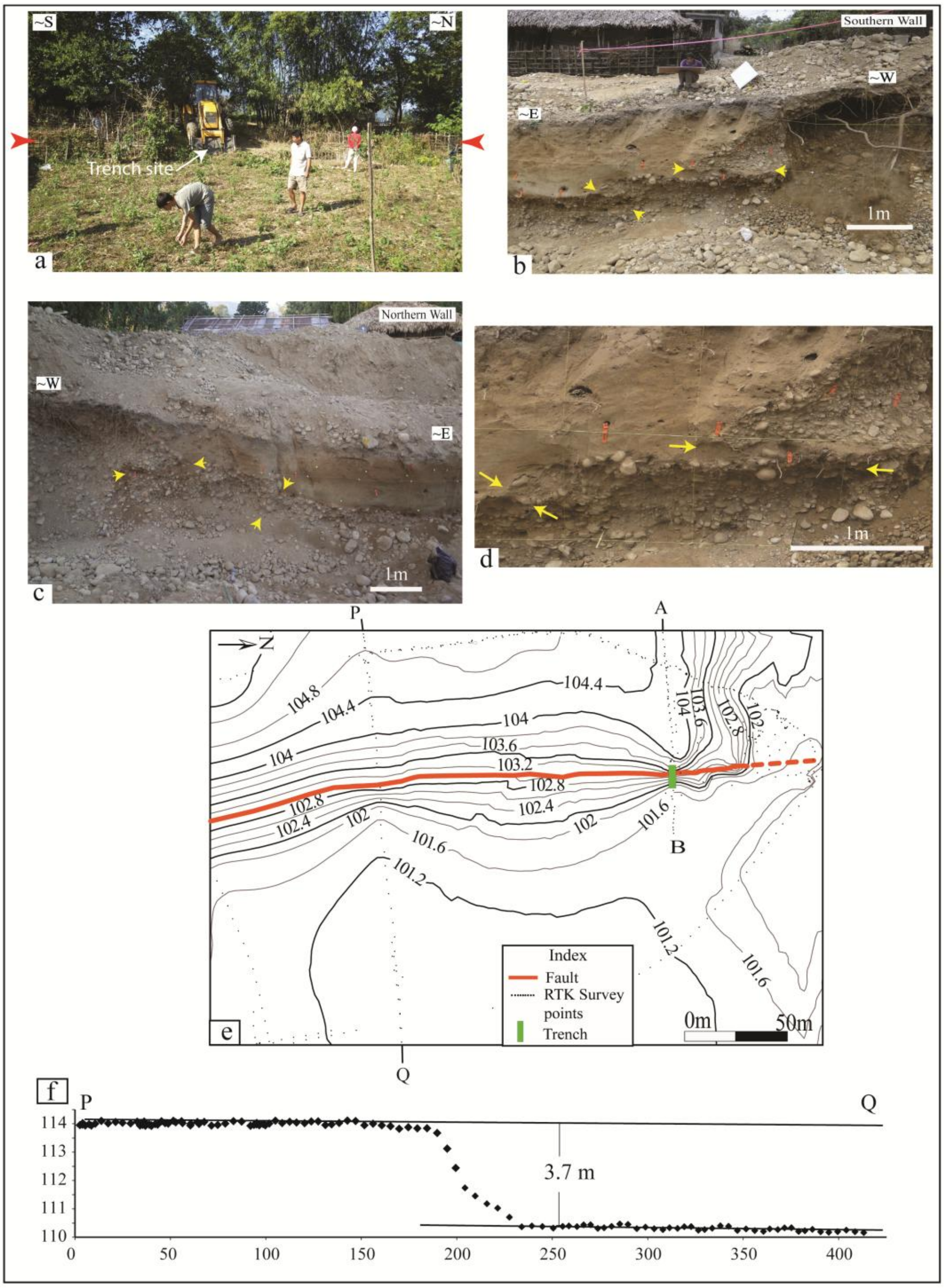

Figure S1. (a) Field photograph of the Pasighat trench site showing the fault scarp; red arrows indicate the base of the scarp. Photos of **(b)** southern and **(c)** northern wall of the excavated trench exposures. Yellow arrows indicate the fault strands exposed in the respective trench walls. **(d)** Magnified image of the southern wall of the excavated trench exposure, yellow arrows indicate the fault strands. **(e)** Micro-topographic map of Pasighat, contours on map are at 0.4 m interval based on survey using Real Time Kinematic Global Positioning System (RTK-GPS). Green bar denotes the location of E-W oriented trench. E-W profile along AB is shown in Fig. 2d and profile PQ is in Fig. S1f. **(f)** RTK-GPS profile of the Pasighat prepared in Leica Geo Office v7.0 programme and artwork in Adobe Illustrator CS5 software.

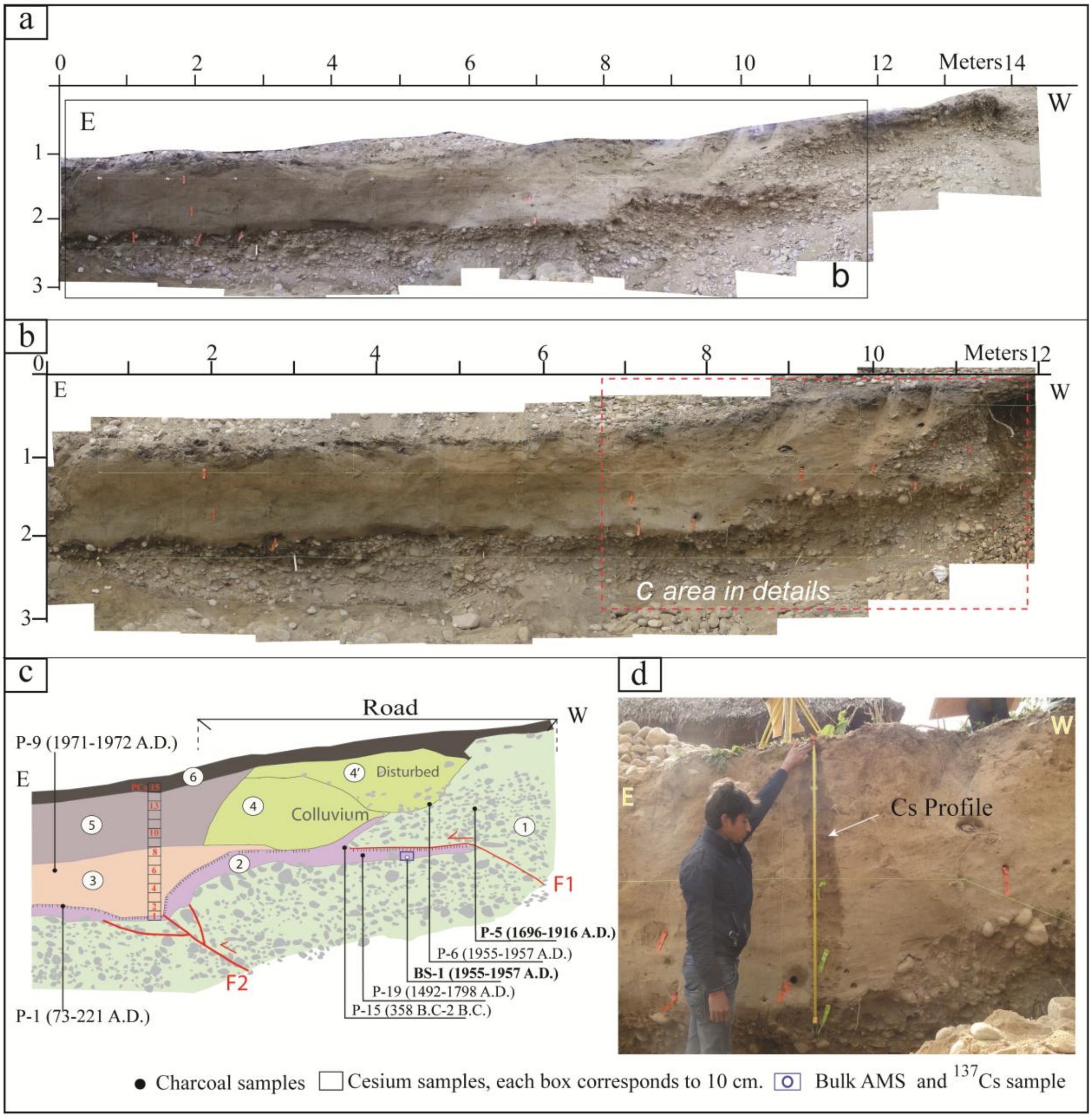

Figure S2. (a) Complete photographic log of the southern wall of the Pasighat trench. Black box is the area shown in Fig. S2b. **(b)** Photomosaic used for the preparation of structural and stratigraphic log shown in Fig. 3c. Red dotted box represents the area showing **(c)** different units and structures of the trench exposure along with the ^{137}Cs profile. Box with number corresponds to samples PCs-1 through PCs-15. **(d)** Photo showing the Cs vertical profile obtained from the Pasighat trench exposure. Unit description of trench log is same as described in Fig. 3c. Mosaic of the trench photographs was done in Adobe Photoshop CS5 software and artwork in Adobe Illustrator CS5.

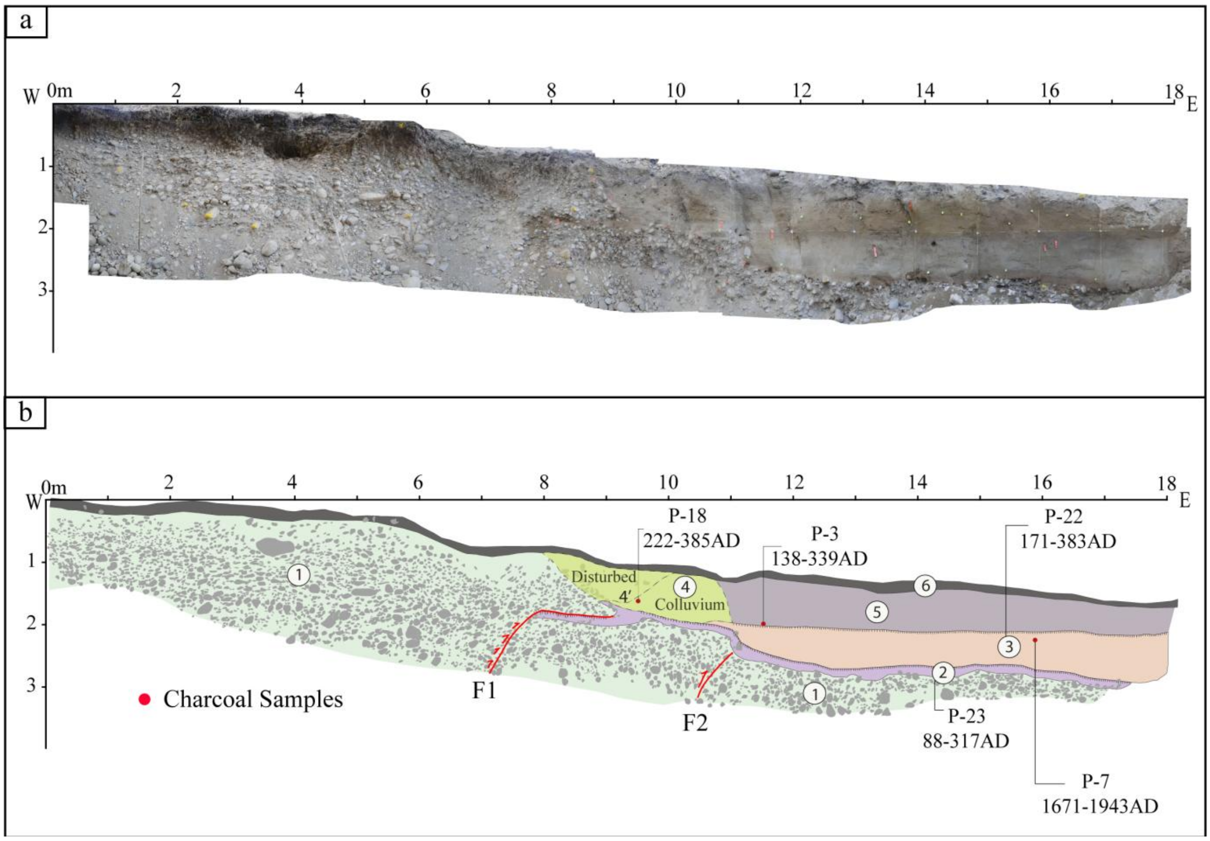

Figure S3. (a) Complete photographic log of the northern wall of the Pasighat trench. **(b)** Illustrative log of the Pasighat trench northern wall showing the structural and stratigraphic units; no vertical exaggeration. Numbers in white solid circles denote the major stratigraphic units discussed in Fig. 3c. Red lines are fault strands exposed in the trench wall denoted as 'F1' and 'F2'. Solid black dots denote the locations of radiocarbon samples with their calibrated ^{14}C AMS ages in calendar year (B.C. or A.D.). Horizontal and vertical scales are the same. Mosaic of the trench photographs was done in Adobe Photoshop CS5 software and artwork in Adobe Illustrator CS5.

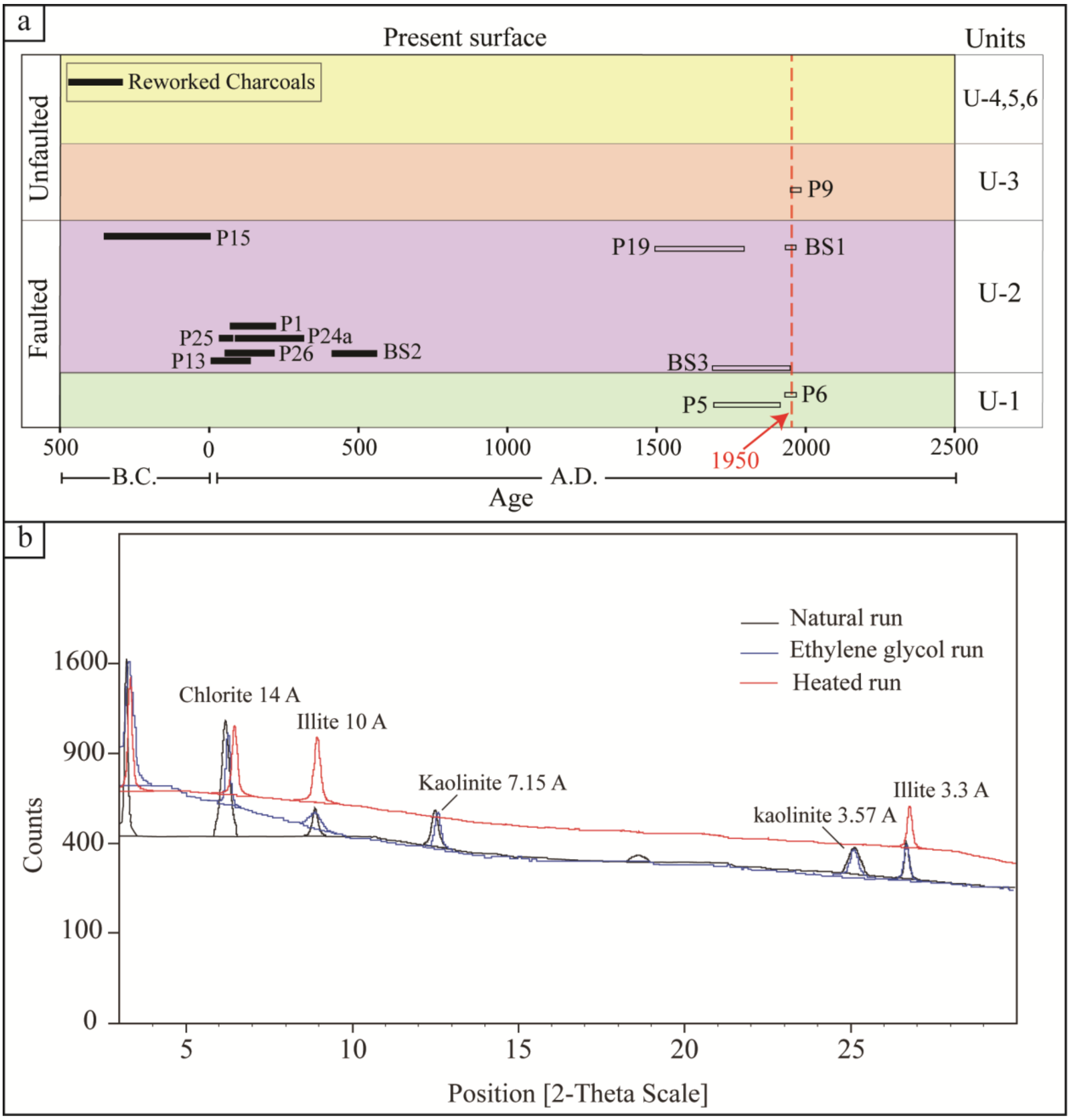

Figure S4. (a) Event horizon diagram showing the stratigraphic position of charcoal samples in the Pasighat trench. **(b)** Interpretation of X-ray diffraction diagrams derived from the three classical runs, i.e., in natural conditions, after ethylene glycol solvation and after 500 °C heating for the clay mineralogy of different stratigraphic units of the Pasighat trench exposure. Figure has been generated by the XRD instrument ‘X’Pert PRO and artwork was done in Adobe Illustrator CS5.

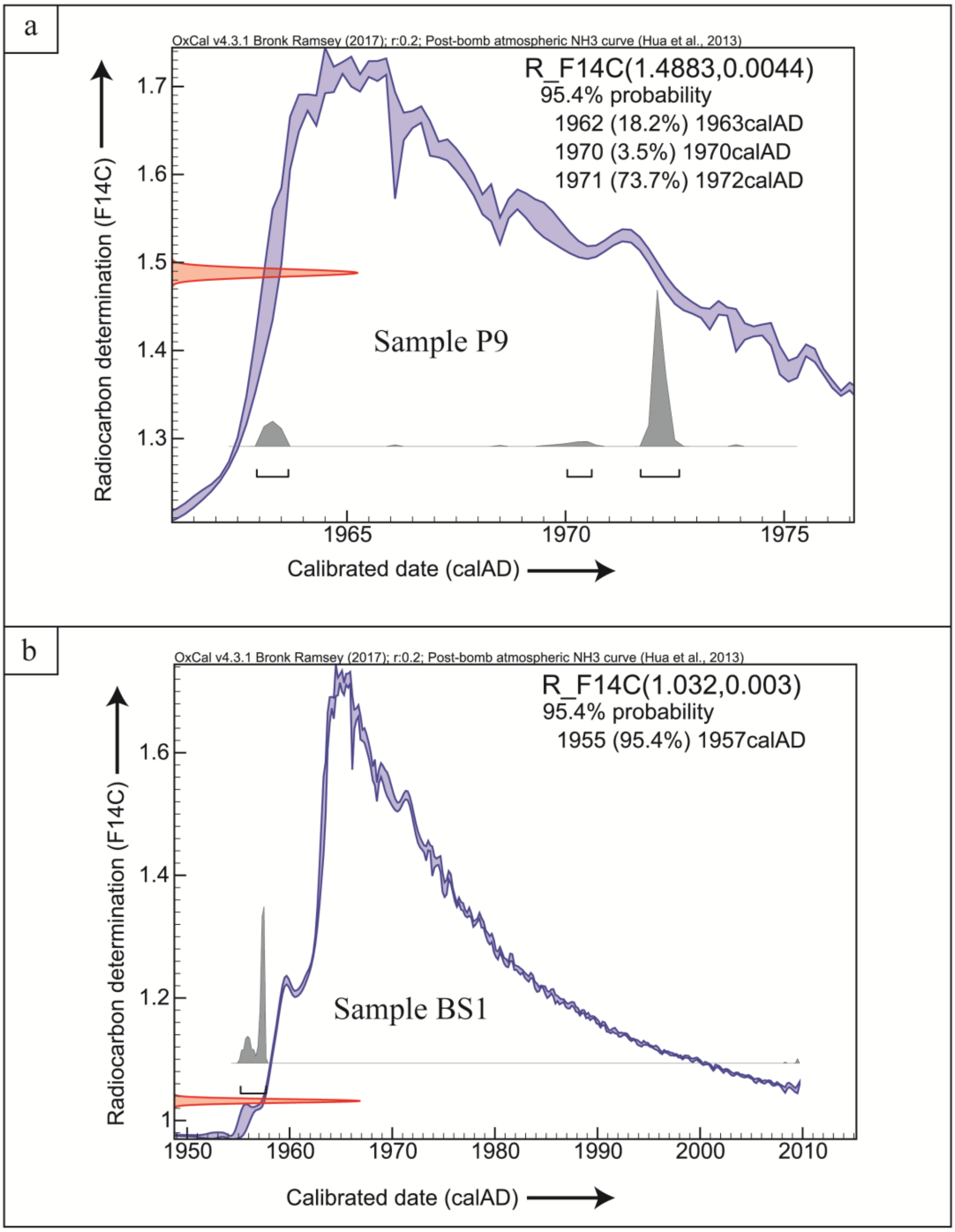

Figure S5. Probability Density Function (PDF) plots of two modern samples obtained from the Pasighat trench southern wall. Sample P9 belongs to unit-3 and BS-1 belongs to the unit-2 (Palaeosol) refer to Fig. 3c. Plots generated by OxCal v4.3.1 online software.

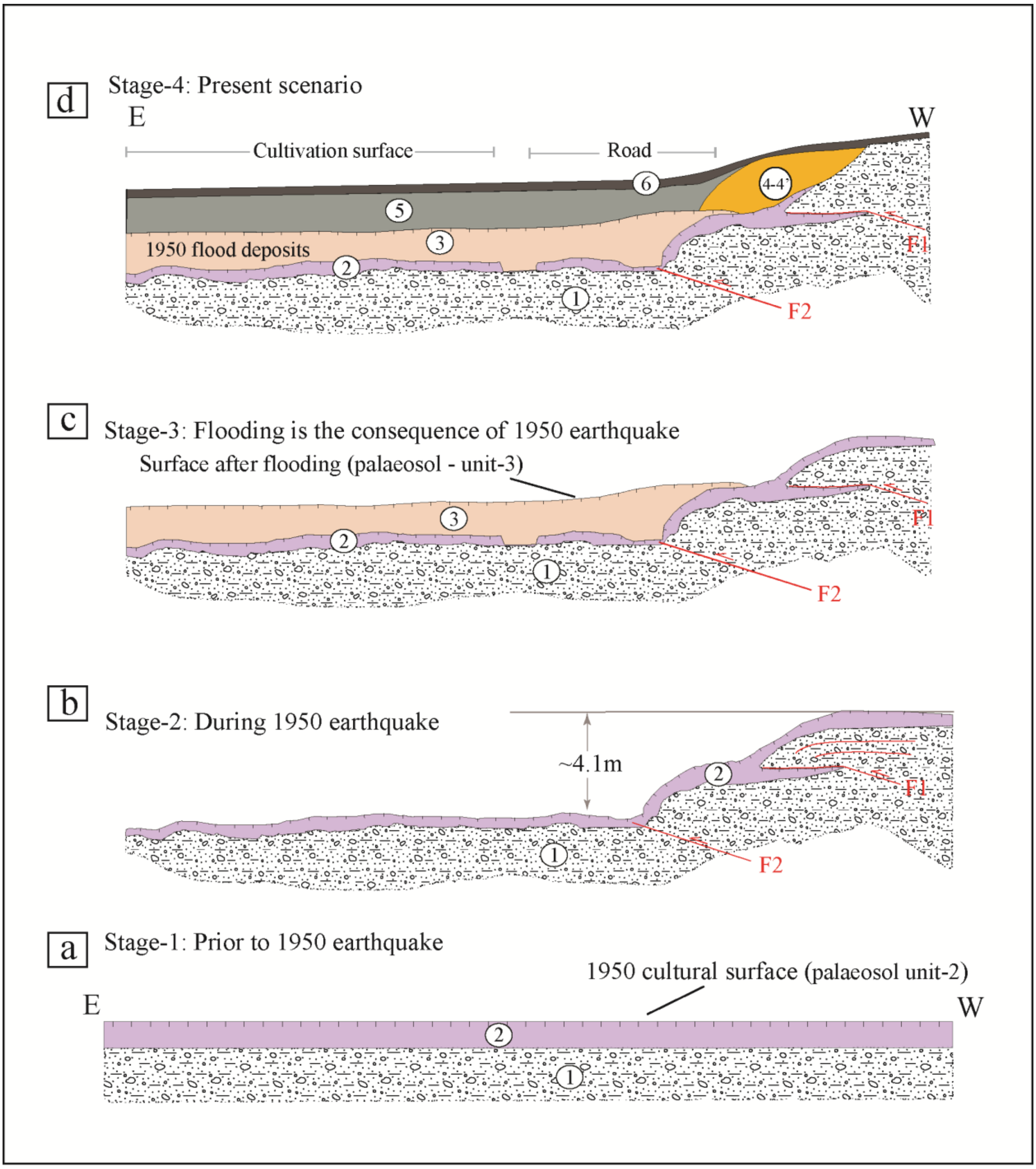

Figure S6. Schematic sketches showing surface faulting stages of the 1950 earthquake along F1 and F2 fault strands and associated flooding.

Progressive stages of deformation of stratigraphic units observed in the Pasighat trench.

Stage-1: Prior to earthquake, the surface was exposed to the atmosphere, receiving the fallout isotopes of pre-1950 in the sediments of unit-2 by diffusion of aerosols (Fig. 5a). Simultaneously, reworked charcoals derived from adjoining higher surfaces were emplaced in unit-2 by surface process.

Stage-2: During the 1950 earthquake, unit-1 (A.D. 1696-1916) and palaeosol unit-2 (A.D. 1945-1950) were deformed; the older unit-1 was emplaced over the palaeosol unit-2 by the faults 'F1' and 'F2'.

Stage-3: Following surface faulting by the 1950 earthquake, the unit-3 was aggraded by a flood event[28]. The flood deposits (unit-3) aggraded following the topography of the fault scarp, which was reduced in height from ~4.1 to 3.1 m.

Stage-4: Progressively, the fault scarp-derived colluvium (unit-4) and the growth stratigraphy (unit-5) were deposited. The units-4, 4' and 5 were modified by bioturbation and anthropogenic activities like plantation and unmetalled road construction.

> A reconnaissance flight over the devastated area was arranged with the I.A.F., but this had to be postponed owing to heavy clouds and poor visibility. This flight may be taken up in early November, when it is expected that the weather in the hills will have improved.
> Except for the Pasighat area and the hilly belt (comprising the Dafla, Abor and a major portion of Mishmi hill ranges), the whole of the Brahmaputra valley was examined during the last six weeks. However, the above remaining portions, which were inaccessible at that time, will be taken up in early November.

Figure S7. Excerption from the post-earthquake damage report of the Geological Survey of India by M.C. Poddar, November, 1950[63].
